# Once Upon a Mine: The Legacy of Uranium on the Navajo Nation

**DOI:** 10.1289/ehp.122-A44

**Published:** 2014-02-01

**Authors:** Carrie Arnold

**Affiliations:** **Carrie Arnold** is a freelance science writer living in Virginia. Her work has appeared in *Scientific American*, *Discover*, *New Scientist*, *Smithsonian*, and more.

On a low, windswept rise at the southeastern edge of the Navajo Nation, Jackie Bell-Jefferson prepares to move her family from their home for a temporary stay that could last up to seven years. A mound of uranium-laden waste the size of several football fields, covered with a thin veneer of gravel, dominates the view from her front door. After many years of living next to the contamination and a litany of health problems she believes it caused, Bell-Jefferson and several other local families will have to vacate their homes for a third round of cleanup efforts by the U.S. Environmental Protection Agency (EPA).

Decades of uranium mining have dotted the landscape across the Navajo Nation with piles of contaminated mine waste. The EPA has mapped 521 abandoned uranium mines on the reservation, ranging from small holes dug by a single prospector into the side of a mesa to large commercial mining operations.^1^ The Navajo people did not have a word for “radioactivity” when mining outfits looking for vanadium^2^ and uranium^3^ began moving onto their land in the 1940s, and they did not understand that radiation could be dangerous. They were not told that the men who worked in the mines were breathing carcinogenic radon gas and showering in radioactive water, nor that the women washing their husbands’ work clothes could spread radionuclides to the rest of the family’s laundry.

Bell-Jefferson and her brother Peterson Bell played in and around the mines, splashing and swimming in pools of radioactive water that had been pumped out of the mines and then collected on their property. The contaminated water looked and tasted perfectly clean. Families used it for cooking, drinking, and cleaning. *Hogans* and corrals were built with mine wastes, as were roads.

All that changed on 16 July 1979. Just about a mile and a half from Bell-Jefferson’s home, a dam broke at the United Nuclear Corporation mill, where workers processed ore from the nearby Northeast Church Rock uranium mine. The spill dumped 94 million gallons of mill process effluent and 1,100 tons of tailings—an acidic, radioactive sludge—into a large arroyo that emptied into the Puerco River.^4^

The Church Rock spill occurred less than four months after the partial meltdown of the Three Mile Island nuclear reactor, and it released three times as much radiation, making it the biggest nuclear spill in U.S. history, yet it received only a tiny fraction of the news coverage.^5^ Declared a Superfund site in 1983, the heaps of waste around the mill still cause radiation survey instruments to squeal from the invisible uranium atoms that remain active 30 years later.^6^

“This area used to be my playground,” Bell-Jefferson says. “Now it’s just a huge wound.”

For the Bells and other Diné (the term by which many Navajo people refer to themselves), the Church Rock spill was a turning point. When corporate and government officials appeared in the spill’s aftermath and began inquiring into exposure to the slurry and potential health problems, the Navajo people finally learned the truth—far from being harmless, these uranium mines were poisoning people, and researchers say they will continue to do so for decades to come.

## Canaries in the Uranium Mines

The arrival of prospectors signified the Navajo Nation’s entrance into the modern wage economy.^7^ Some welcomed the potential income. In 1995 former uranium miner George Tutt recollected, “We were blessed, we thought. Railroad jobs were available only far off like Denver. … But for mining, one can just walk to it in the canyon. We thought we were very fortunate, but we were not told, ‘Later on this will affect you in this way.’”^7^

Yet researchers had noted as early as 1879 that uranium miners in Europe showed significantly elevated levels of lung cancer.^8^ By the 1930s, they suspected radiation as the culprit.^9^ As early as 1951, government scientists had begun to work out what made uranium so deadly. The answer, as it turned out, wasn’t uranium itself but its decay products, including radium,^10^ thorium,^11^ and radon.^12^

Radon is a gas, but with a half-life of four days, it rapidly decays into solid products, explains Doug Brugge, a professor of public health at Tufts University.^13^ “Being solids, these are going to want to stick to things like your lungs,” Brugge says. “Both radon and its daughter products emit alpha particles, and this is a very effective way to cause damage that can lead to cancer.”

In just over a decade, Navajo miners were being diagnosed with lung cancer,^14^ a relatively rare disease in this largely nonsmoking population.^15^ Beginning in 1950, workers with the U.S. Public Health Service led by Duncan Holaday and Victor Archer began following uranium miners in the Southwest, both Navajo and white, to measure their exposures and assess their specific cancer risks. To get access to the workers, the researchers had to strike a Faustian bargain with the mining companies: They could not inform the miners of the potential health hazards of their work.^2^ Seeing it as the only way to convince government regulators to improve safety in the mines, the researchers accepted.^16^ By 1965, the investigators reported an association between cumulative exposure to uranium and lung cancer among white miners and had definitively identified the cause as radiation exposure.^17^

In 1984 another team published results of a case–control study that further implicated uranium mining as a cause of lung cancer in Navajo men. The team analyzed 96 confirmed cancer cases from the New Mexico Tumor Registry, 32 lung cancer cases and 64 cases of other cancers. Of the 32 Navajo men who developed lung cancer, 72% had worked as uranium miners, compared with none of the controls. Furthermore, the median age of miners with lung cancer was 44 years, compared with 63 years for nonminers with other cancers.^18^ Decades after their exposure ended, standardized mortality ratios and relative risks for lung cancer and other respiratory problems were still nearly four times higher in Navajo miners than in nonminers.^19^

## Community Exposure to Uranium

Getting the ore out of the ground was only the first step in a long process. Miners then transported the ore to a mill, where it was crushed and soaked in sulfuric acid to extract the uranium.^20^ More chemicals were added to precipitate out the uranium, leaving behind a radioactive slurry. This slurry was frequently stored in large, unlined ponds, says Chris Shuey, an environmental health specialist with the Southwest Research and Information Center in Albuquerque, who has spent the last three decades working with Navajo communities affected by uranium mining and milling.

Mining in the area had mostly ceased by the mid-1960s. Today, after decades of inactivity, the uranium from these ponds, waste and tailings piles, and the mines themselves is still present in highly chemically soluble forms^6^^,^^21^ that have been leaching into the area’s drinking water, according to water testing by the EPA and the Army Corps of Engineers.^22^

In a small, one-story adobe building tucked into the far edge of the University of New Mexico campus, Johnnye Lewis, a professor of toxicology, has spent more than a decade studying mining-related health effects in the Navajo people. In 2000 she received an environmental justice grant from the National Institute of Environmental Health Sciences to collect clinical and survey data from people living on the Eastern Navajo Nation. The DiNEH (Diné Network for Environmental Health) Project was originally started to address community concerns about the high rate of kidney disease in this population, which some community leaders and health professionals suspected was related to drinking contaminated water.

Lewis and colleagues surveyed 1,304 residents, obtaining basic demographic information, mapping the locations of their homes, and taking samples from the wells where they obtained their drinking water. Of these, 267 provided blood and urine samples so the researchers could look for markers of biological damage.^23^ The average age of study participants was 51.5 years.

The data the team amassed over the last 13 years suggests that health problems from these mines in fact aren’t limited to the miners who worked in them but also extend to those exposed through drinking water or simply living near a mine. “We’re still analyzing data—it generated just an enormous amount of data,” Lewis says. “But what we will end up with is that we now will be able to study three successive generations of Navajos that have been exposed.”

Although the literature on chronic low-level uranium exposure is still quite small, by 2003 researchers knew that the dangers these exposures posed were due not to uranium’s radioactivity but to its chemical toxicity.^24^ Both animal^25^ and human^26^ studies have found uranium to be primarily toxic to the kidneys. One such study, led by Maria Limson-Zamora, head of Health Canada’s Bioassay Section, compared biomarkers of kidney function in the urine of Canadians chronically exposed to high (2–780 µg/L) or low (0.02 µg/L) levels of uranium in their drinking water. The investigators found signs of kidney damage that increased with higher daily intake of uranium in the drinking water.^27^

Uranium appears to exert its chemical effects on the kidney’s proximal tubules.^28^ Arsenic and cadmium—which, along with other potentially hazardous metals, are sometimes found in uranium tailings^29^—create similar signatures of metal damage in the kidneys.^30^

Lewis’s early data from the DiNEH Project suggest that self-reported kidney disease, hypertension, and autoimmune diseases were more prevalent among people who lived closer to mine waste sites.^31^ Her colleague at the University of New Mexico, immunologist Ester Erdei, believes the increase in hypertension and autoimmune diseases might be connected to consumption of contaminated water.

A growing body of evidence links hypertension,^32^ heart disease,^33^ and autoimmune diseases^34^ to markers of inflammation such as C-reactive protein and assorted chemokines.^35^ Erdei hypothesizes that uranium exposure might contribute to these diseases through effects on inflammation. She recently presented findings showing an association between increased levels of activated T cells in DiNEH Project participants and greater residential proximity to mine waste sites.^36^

“If we see any of these activated T cells, we know that the immune system is highly reacting to something,” Erdei says. “We didn’t know what it is. This is the next step to find out how it’s really happening on the molecular level.”

## Uranium’s Toxic Legacy

Human and animal studies elsewhere have indicated the health legacy of uranium exposure may extend to the children of exposed parents. A study of 266 cases and matched controls among Navajo births over 18 years suggested that children of women who lived near abandoned uranium sites were 1.83 times more likely to have 1 of 33 selected defects. Among these were defects thought to be connected to radiation exposure (e.g., chromosomal disorders, single gene mutations) as well as distinctly nonrelated defects (e.g., deaths due to obstetrical complications). On the other hand, these outcomes also were twice as common among children whose mothers worked at an electronics assembly plant as in other children.^37^

Animal studies suggest potential reproductive implications of exposure. A study in rats exposed to uranium found the offspring had a higher body burden of uranium than the dams. These offspring also had higher rates of physiological changes, including atypical sperm formation.^38^ And a mouse study produced evidence that uranium in drinking water caused estrogenic activity even at levels below the EPA safe drinking water level of 30 µg/L.^39^

To look more closely at the effects of uranium exposure on human reproduction and development, Lewis has recently begun recruiting up to 1,500 pregnant women to participate in the Navajo Birth Cohort Study.^40^ Besides tracking birth outcomes and infant development, pharmacologist Laurie Hudson of the University of New Mexico is looking at molecular changes that may be induced by exposure to uranium waste.

Arsenic is chemically very similar to zinc and can replace zinc in proteins that are important in DNA repair.^41^ “Arsenic goes in and kicks zinc out, but the arsenic doesn’t replace the function of zinc. So the proteins become incapacitated,” Hudson says. This creates a hat trick of DNA damage: Uranium’s radioactive^42^ and chemical^43^ properties both can harm DNA, and the presence of arsenic may prevent cells from repairing the damage.

Animal and cell culture studies have suggested a potential solution: zinc supplementation.^44^ Hudson and Lewis want to see if zinc supplementation may prevent arsenic from damaging DNA repair enzymes in women enrolled in the Navajo Birth Cohort Study, and they have identified an easy way to do this. Prenatal vitamins, which contain zinc, are generally obtained via a prescription through the Indian Health Service. Researchers can determine which women are taking their vitamins by who refills their prescription. Women who don’t take vitamins will serve as the control group. The investigators will have information on the women’s environmental exposures and their body burden of metals, so they can start to zero in on how arsenic and uranium exposures affect protein function and whether zinc supplementation provides any protection.

The findings will provide a concrete way for the researchers to give back to the community. “We’ve pretty much been clear from the beginning that if we see something that’s wrong, we’re not going to let it stick around just to preserve the data,” Lewis says. “We’re going to make sure people know their risks and can take action.”
